# Osseous vitality in single photon emission computed tomography/computed tomography (SPECT/CT) after balloon tibioplasty of the tibial plateau: a case series

**DOI:** 10.1186/s12880-015-0091-y

**Published:** 2015-11-17

**Authors:** Thorsten Jentzsch, Yannick Fritz, Patrick Veit-Haibach, Jürgen Schmitt, Kai Sprengel, Clément M. L. Werner

**Affiliations:** Division of Trauma Surgery, Department of Surgery, University Hospital Zurich, Ramistrasse 100, 8091 Zurich, Switzerland; Division of Nuclear Medicine and Diagnostic and Interventional Radiology, Department of Medical Radiology, University Hospital Zürich and University of Zurich, Zurich, Switzerland

**Keywords:** Osseous vitality, SPECT/CT, Balloon-assisted, Cement-augmented, Tibioplasty, Proximal tibia

## Abstract

**Background:**

The minimally invasive, balloon-assisted reduction and cement-augmented internal fixation of the tibial plateau is an innovative surgical procedure for tibial plateau fractures. The close proximity of balloons and cement to the knee joint poses a potential risk for osteonecrosis; especially in the case of thin bone lamellae. However, there are no studies about the vitality of the cement-surrounding tissue after these tibioplasties. Therefore, our goal was to assess the osseous vitality after cement-augmented balloon tibioplasty using single photon emission computed tomography/computed tomography (SPECT/CT) in a series of patients.

**Methods:**

This case series evaluated available consecutive patients, whose tibial plateau fractures were treated with balloon-assisted, cement-augmented tibioplasty and received a SPECT/CT. Primary outcome variables were osseous vitality on SPECT/CTs according to the semiquantitative tracer activity analysis. The mean uptake of eight tibial regions of interest was referenced to the mean uptake count on the same region of the contralateral leg to obtain a count ratio. Osteonecrosis was defined as a photopenic area or cold defect. Secondary variables included clinical and radiological follow-up data. Statistics were carried out in a descriptive pattern.

**Results:**

Ten patients with a mean age of 59 years and a mean follow up of 18 months were included. Calcium phosphate (CaP) substitute bone cement was used in 60 % and polymethyl methacrylate mixed with hydroxyapatite (PMMA/HA) bone cement in 40 %. Normal to high SPECT/CT activity without photopenic areas were observed in all patients and the mean tracer activity ratio was four, indicating vital bone in all patients. There were no postoperative infections and only one 57 year old patient with hemineglect and CaP cement showed failed osseous consolidation. The mean Tegner and Lysholm as well as the Lysholm scores were three and 80, respectively.

**Conclusions:**

This novel study about cement-augmented balloon tibioplasties showed that osseous vitality remains intact according to SPECT/CT analysis; irrespective of the type of cement and even in the presence of thin bone lamellae. This procedure was safe and well-suited for lateral tibial plateau fractures in particular. Surgeons may consider using PMMA/HA bone cement for void filling in elderly fracture patients without concern about bone viability.

## Background

Fractures of the tibial plateau account for 2 % of all fractures, 8 % of fractures in the elderly with a peak incidence in the fifth decade, and 10 % of tibial fractures [1–3]. They show a bimodal age distribution, where fractures in the young are often caused by high-energy injuries such as motor vehicle collisions or skiing accidents, whereas fractures in the elderly are mostly of osteoporotic nature caused by falls [4]. The operative management of displaced fractures is difficult due to common soft tissue compromise in more than ^2^/_3_ of these fractures and fracture comminution giving way to consecutive risks of infection, post-traumatic arthritis, anatomical misalignment, and nonunion [3, 5]. The surgical goal is to achieve anatomical reduction, restoration of articular congruency with step-offs preferably under two millimeters, and primary stability for early functional rehabilitation [6, 7]. The gold standard is open reduction and internal fixation with a locking plate, which can also be inserted percutaneously and unilateral on the lateral side with angular stability to reduce the infection rate to under 10 % [8, 9].

The minimally invasive, balloon-assisted reduction and cement-augmented internal fixation of the tibial plateau, also termed balloon tibioplasty, is an innovative, possibly superior surgical procedure [7, 10]. Originally designed as a kyphoplasty for vertebral compression fractures, it uses a trocar to enter the bone, a balloon to dilate and reduce, and cement for augmentation [11]. Its elegance lies in the indirect elevation of the impressed defect instead of mechanical elevation with regular instruments, which may even enlarge the void created by the imprecise reducing action [12]. It has been shown to have similar reduction capabilities as conventional mechanical tamps and can be used successfully to elevate tibial impressions [13]. Another advantage of this technique consists of the percutaneous approach without periosteal stripping/fenestration, which means a reduction in postoperative complications such as infections, delayed wound healing and pseudarthrosis [14]. Although the cement is rather expensive, it is still cheaper than other synthetic bone substitutes. The procedure with polymethyl methacrylate (PMMA) may be followed by immediate full weight-bearing with shorter hospitalization times as well as faster rehabilitation, less postoperative infections, and, therefore, reduced overall costs [10]. In the beginning, the indication for balloon tibioplasties were elderly, multimorbid patients at risk of postoperative wound complications and in need of prompt rehabilitation [10]. Recently, due to the increasing popularity of calcium phosphate (CaP) cements, these procedures have also been expanded to younger patients and hardly accessible posterolateral impression fractures, which can be computed tomography (CT)-navigated in difficult cases. In this context, the effect of cement-induced heat has been studied in kyphoplasties at the spine, but varying results have been reported [15, 16]. Up-to-date, there are no studies about the effects of cement augmentation of tibial plateaus on bone viability, and especially not after inflatable bone tamp reduction as used in the technique of balloon tibioplasty. However, this is of particular interest because of the close proximity of balloons and cement to the joint, which poses a potential risk for osteonecrosis; especially in thin bone lamellae.

Single photon emission computed tomography (SPECT) delivers a highly sensitive 3-dimensional (3-D) image through varying intensity of emitted gamma rays based on the reactive changes and metabolism of a gamma-emitting radioisotope, technetium (Tc) [17, 18]. Superimposed with highly specific CT, which accurately depicts the anatomical morphology, it is particularly useful for the direct detection of osseous vitality [19]. Although this imaging modality has been used in spinal, pelvic, foot and ankles injuries [20], there are is a lack of studies about the vitality of the cement-surrounding tissue after balloon tibioplasty of the knee. Therefore, this study aimed to assess the osseous vitality after cement-augmented balloon tibioplasty using SPECT/CT in a series of patients.

## Methods

**Fig. 1:**
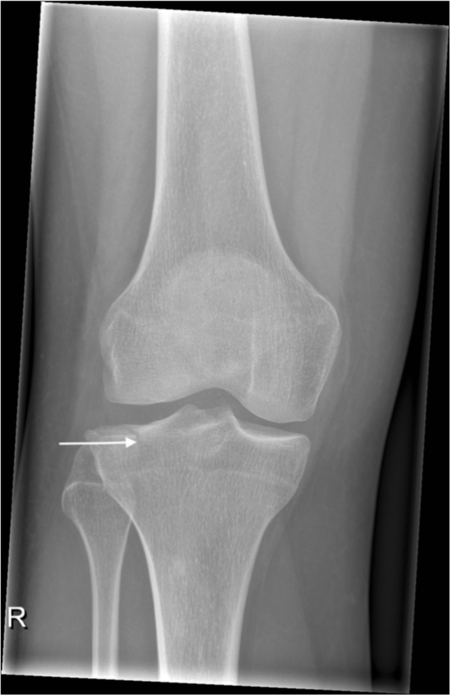
Anteroposterior X-ray view of a tibial plateau fracture (Schatzker II) with a white arrow indicating the upper part of the fracture, which led to a split, depression > 2 mm and loss of convexity of the lateral tibial plateau

**Fig. 2:**
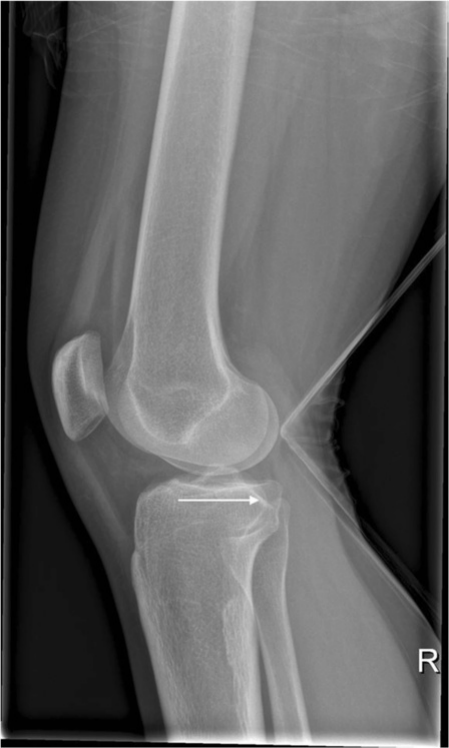
Lateral X-ray view of a tibial plateau fracture (Schatzker II) shown in Fig. 1

**Fig. 3:**
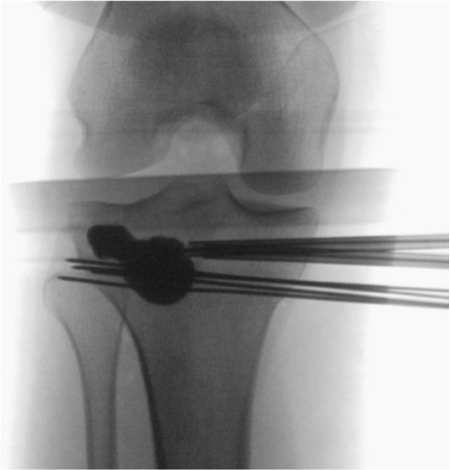
Intraoperative anteroposterior X-ray view of a minimally invasive, balloon-assisted reduction and cement-augmented internal fixation of a tibial plateau. Since the first balloon evaded distally, four Kirschner wires were used to buttress two balloons to cause the elevation of the impression and the final, successful reduction

**Fig. 4:**
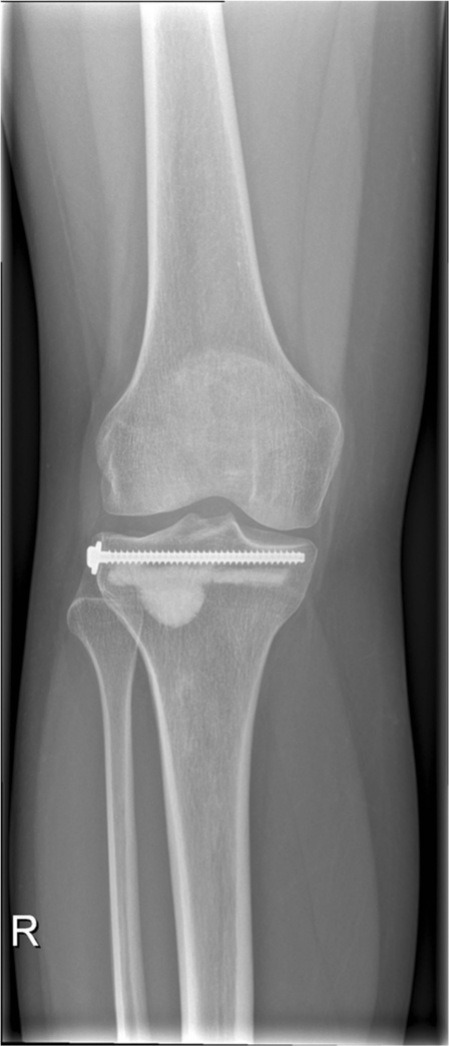
Anteroposterior X-ray view of a successful cement-augmented tibioplasty of a tibial plateau fracture (Schatzker II)

**Fig. 5:**
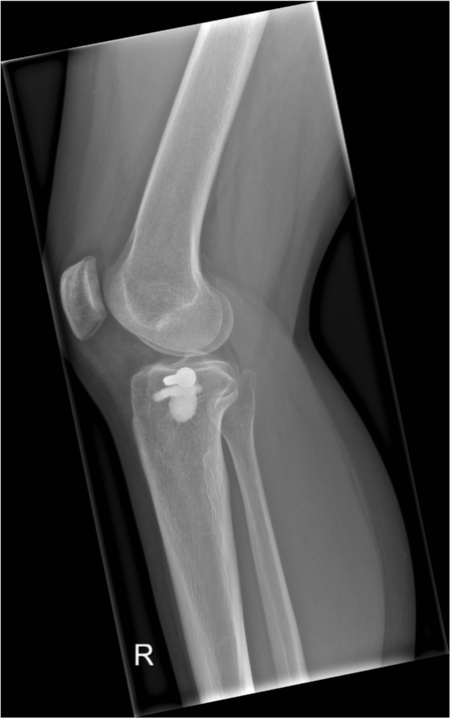
Lateral X-ray view of a successful cement-augmented tibioplasty of a tibial plateau fracture (Schatzker II)

**Fig. 6:**
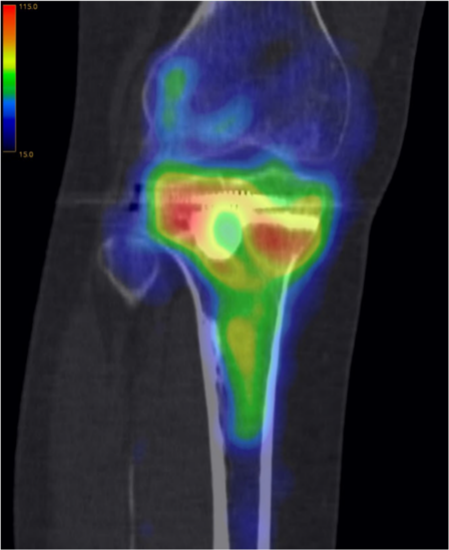
A coronal view of a SPECT/CT is shown 5 months after balloon tibioplasty. The blue to red colors show tracer activity in an increasing order and the normal to high tracer activity is particularly located around the cement and the osteosynthesis. In contrast, no color and no tracer activity can be found in the bone marrow of the tibial shaft

**Fig. 7:**
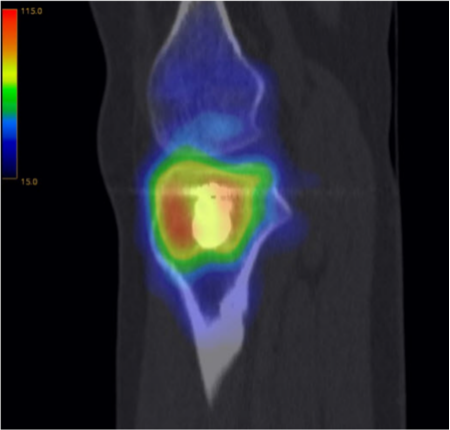
A sagittal view of a SPECT/CT corresponding to Fig. 6 is shown

**Fig. 8:**
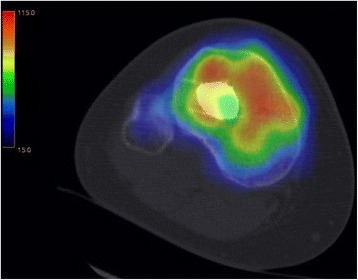
An axial view of a SPECT/CT corresponding to Figs. 6 and 7

**Fig. 9:**
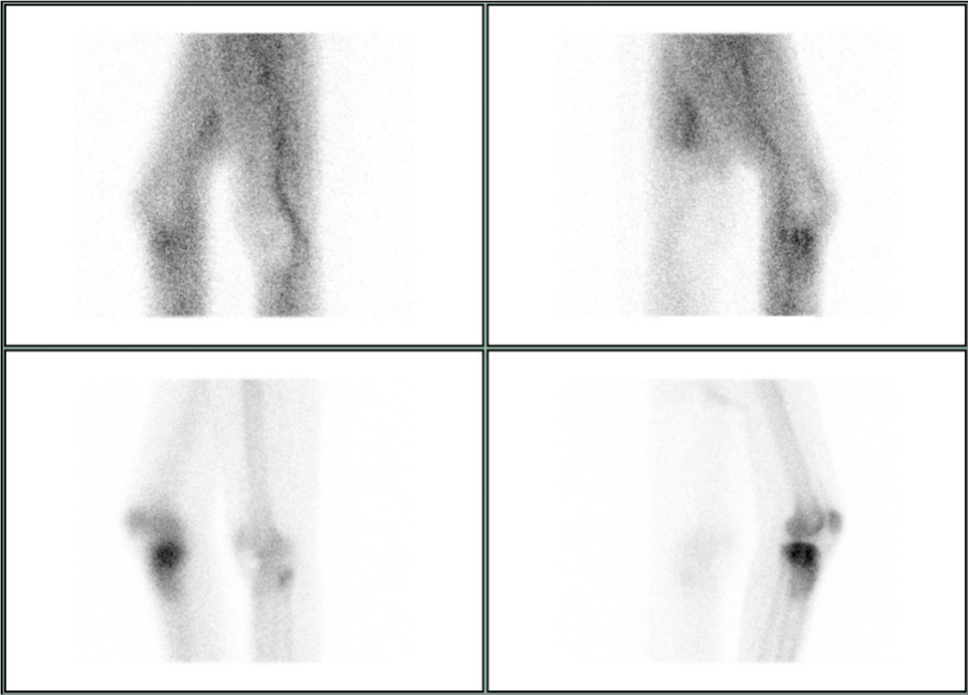
A SPECT/CT corresponding to Figs. 6-8 is shown with early (top) and late (bottom) phases of anteroposterior (left) and lateral (right) views

This study at a level one trauma center evaluated available consecutive patients, whose tibial plateau fractures were treated with a balloon-assisted, cement-augmented tibioplasty between 2011 and 2014 (Figs. 1, 2, 3, 4 and 5). Of 16 patients with balloon tibioplasty, ten patients received SPECT/CT (Figs. 6, 7, 8 and 9). This is an option to assess osseous viability aside from routine radiological imaging with plain films or CT scans for assessment of fracture healing and consolidation. Reasons for loss to follow up were death (*n* = 2), opting out due to unreasonable expenditure in the elderly (*n* = 3), and moving abroad (*n* = 1). This study expands a previous report by the authors' group [10], which included three of the patients in this study and exclusively described the methods of this innovative surgical technique without focusing on the clinical outcomes or osseous vitality. The primary outcome variable was vital bone on SPECT/CT based on the tracer uptake. Osteonecrosis was defined as a photopenic area or “cold defect”. Secondary outcome influencing variables included the fracture type, cement and osteosynthesis type, surgical duration, hospital stay duration, infection, wound healing, pain at last-follow up, ability to work at last follow-up, osseous consolidation, and two knee-specific scores. This study was approved by the cantonal ethics committee (KEK-ZH-Nr. 2014–0557) and only included adults over 18 years.

As previously described [18], SPECT/CT (DiscoveryNM/CT 670; GE Healthcare, Waukesha, WI, USA) included a standardized pre-interventional injection of 650–700 Megabecquerel (MBq) ^99m^Tc-3,3-diphosphono-1,2-propanodicarboxylic acid (^99m^Tc-DPD) (Teceos™; IBA Molecular, Louvain-la-Neuve, Belgium). It used three phases, a planar perfusion phase right after the injection lasting 3 minutes followed by an early blood pooling phase lasting 7 minutes, and a late phase after 3 hours, ultimately providing spot and CT images with a slice thickness of 1.25 mm in axial, sagittal, and coronal planes. These images were then merged with an automatic software algorithm (Advantage Workstation 4.5; GE Healthcare, Waukesha, WI, USA). The tracer activity was documented semiquantitatively by an experienced orthopaedic surgeon after thorough instruction, under supervision, and in consensus with a nuclear medicine attending physician/radiologist. It was displayed in the form of a standard thermal color scale. For the semiquantitative analysis, the tracer activity was recorded on a modified standardized localization scheme originally developed for patients after total knee arthroplasty [21]. Surrounding the axial and coronal slices with the highest amount of cement, eight tibial regions of interest (ROIs) with a standard area of nine pixels were selected on a workstation (Xeleris™, Version 3.0513; General Electric Company, Fairfield, CT, USA). They consisted of an antero-lateral, −median, −medial, a postero-lateral, −median, −medial as well as a supero-median, infero-median region. Then, the mean uptake count (counts per pixel) was automatically defined [22]. This mean uptake was then referenced to the mean uptake count on the same region of the contralateral leg to reach a count ratio. An uptake count ratio of 1 was termed normal and a ratio of ≥ 2 was named high.

The fracture type was classified according to Schatzker [23]. Furthermore, the types of cement type and osteosynthesis were recorded as well. KyphOs FS™ (Medtronic Inc., Fridley, MN, USA) is a CaP substitute bone cement and Kyphon® ActivOs™ (Medtronic Inc.) is a mixture of PMMA and hydroxyapatite (PMMA/HA) bone cement. Patients were either treated with an additional plate or screw osteosynthesis. Further variables consisted of the continuous variables of the surgical duration [minutes] and duration of the hospital stay [days] as well as categorical variables of infection, wound healing, pain at last-follow up, ability to work at last follow-up, and osseous consolidation. Moreover, two knee-specific scores, i.e. the Tegner and Lysholm score [24] (0–10 points) and the Lysholm score [25] (0–100 points) were obtained.

All data were obtained from thorough chart and radiologic review before being stored in an Excel (Microsoft Corp., Redmond, WA, USA) spreadsheet. They are presented as means with their standard deviation (SD). Statistical analysis was carried out in a descriptive pattern due to the rather small sample size.

## Results

The mean age of the ten patients included in this study was 59 (SD 22) years and there were more females (*n* = 9) than males (*n* = 1) at a mean follow up of 18 (SD 14) months (table 1). The bone was vital in all patients. No signs of osteonecrosis were observed at different times during the course of treatment; neither after two months, nor after more than three years. The osseous vitality of one patient was evaluated after a knee prosthesis had been implanted. The SPECT/CT activity around the cement was normal or high in all patients. No photopenic areas or cold defects were observed. The mean tracer activity of the cement-surrounding tissue was 525 (SD 367), while the mean tracer activity of the contralateral side was 163 (SD 69). Therefore, the mean tracer ratio was 4 (SD 3).

**Table 1:** Patient characteristics

Patient	Age (y)	Sex	Schatzker	Follow-up (m)	SPECT/CT tissue vitality			Cement	Hardware	Surgery (min)	Hospital stay (d)	Infection	Delayed wound healing	Pain at rest	Osseous consolidation	Pre- to-post-operative degenerative changes	Regained pre-operative ability to work	Tegner and Lysholm score	Lysholm score
					Bone	Tracer mean	Tracer ratio												
1	53	F	II	37	Y	170	1	K	P	110	10	N	N	N	Y	N	Y	5	95
2	85	M	II	35	Y	493	3	A	P	150	18	N	N	N	Y	N	NA	1	53
3	57	F	V	33	Y	365	3	K	P	75	13	N	N	N	N	N	NA	1	53
4	85	F	IV	33	Y	361	1	A	P	120	28	N	N	N	Y	N	NA	3	70
5	64	F	V	15	Y	325	2	A	P	120	12	N	N	N	Y	N	NA	3	85
6	80	F	V	11	Y	402	3	A	P	135	14	N	N	N	Y	N	NA	3	85
7	68	F	II	7	Y	661	4	K	P	130	13	N	N	N	Y	N	NA	3	86
8	30	F	II	5	Y	666	6	K	S	90	5	N	N	N	Y	N	Y	4	85
9	33	F	II	5	Y	527	4	K	P	90	9	N	N	N	Y	N	Y	4	91
10	31	F	II	2	Y	1284	9	K	S	70	2	N	N	N	Y	N	Y	4	95

(y) years, (m) months, (SPECT/CT) single photon emission computed tomography/computed tomography, (min) minutes, (d) days, (F) female, (Y) yes, (K) KyphOs FS, (P) plate, (N) no, (M) male, (A) ActivOs, (na) not applicable, and (S) screw

The majority of patients (*n* = 6) sustained a Schatzker II fracture, while three patients had Schatzker V fractures, and one patient presented with a Schatzker IV fracture (table 1). KyphOs FS™ (Medtronic Inc.) was used in six cases, while Kyphon® ActivOs™ (Medtronic Inc.) was inserted in four patients. Most patients (*n* = 8) had an additional plate fixation, while two patients were treated with additional subchondral, latero-medial screws. The mean surgical time was 109 (SD 27) minutes. There were no intraoperative perforations into the joint. The mean length of hospital stay was 12 (SD 7) days. There were no cases of postoperative infection or delayed wound healing. The majority of patients (*n* = 9) reported no pain at rest, but at heavy exercise and one patient was completely pain-free at all times. All patients regained their pre-operative ability to work. Osseous consolidation was achieved in a mean time of 2 (SD 1) months in all but one patient with hemineglect, osteopenia and CaP cement, who showed a loss of reduction with a painfree functional deficit and received a total knee arthroplasty 4 months postoperatively. There were no pre- to post-operative degenerative changes. The mean Lysholm scores was 80 (SD 16) and the mean pre- and postoperative Tegner and Lysholm scores of 3 (SD 1) stayed the same.

## Discussion

This study represents the first investigation of the vitality of the cement-surrounding tissue using the example of balloon tibioplasty of the proximal tibia. According to the SPECT/CT findings, the osseous vitality of the tibial plateau remains intact after minimally invasive, balloon-assisted reduction and cement-augmented internal fixation; independently of using PMMA/HA or CaP cement and even with close proximity of the balloon and cement to thin bone lamellae. Despite an acceptable clinical outcome without postoperative infections and a mean Lysholm score of 80, one failed osseous consolidation was observed in a patient with CaP cement and hemineglect.

In the beginning of the 20th century, the basic materials for bone cements, which include the polymerization of methacrylates, nowadays supplied in the form of a two-component system with mixable powder and liquid, were first introduced by Otto Röhm [26]. In the field of orthopaedic surgery, cement was first used by John Charnley for the anchorage of the femoral component of a hip endoprosthesis in the 1958 [27]. Polymethyl methacrylate cement is not integrated into bone tissue, but, instead, separated by a fibrous membrane [28]. Contrarily, CaP cement is osteoinductive and –integrative, which may eventually lead to substitution by autologous bone apposition. Their drawback lies in the lack of intrinsic structural integrity, which leads to their mechanical instability until complete tissue ingrowth. Furthermore, PMMA cement hardens under heat-production through an exothermic reaction, while CaP cement crystalizes with a slower reaction and less thermal production [29–34]. In the spine, it was shown that PMMA may lead to intravertebral thermal necrosis without affecting structures around the vertebral body [31]. In cadaveric vertebral bodies and the adjacent spinal canal, temperatures ranged from 44–112 and 39–57 °C, respectively, while dwell times at temperatures higher than 50 °C lasted up to 8 and 2.5 min, repsectively [35]. Another in vitro study showed smaller temperatures, but they were still above 50 °C [36]. A different in vitro study showed temperature drops from the center of cement to the cement/bone interface with no thermal necrosis when augmenting hip screws with 6 cc cement despite potential risks of thermal necrosis with PMMA layers larger than 5 mm [37]. However, it remained unknown whether possible surrounding tissue necrosis may be attributed to heat or other factors and whether it may spread from the bone into the cartilage of the proximal tibia. Our findings are in line with the experimental results by Baroud et al. [32], who concluded that neither PMMA nor CaP cements produce enough heat to cause excessive thermal injury, although they led to temperature increases of 23° and 4 °C.

In contrast to magnetic resonance imaging (MRI), using SPECT/CT for the evaluation of osseous vitality provided the benefit of pointing out the region of interest by showing increased (or absent) metabolism without displaying abundant and superfluous details [19] in fast examinations with lower risks of claustrophobia. Furthermore, metal implants after osteosynthesis may lead to substantial artifacts in the MRI, while planar images are associated with a lower image resolution. Combining functional SPECT images with high-resolution morphologic CT scans, osteonecrosis can be well evaluated, which has been shown in a SPECT/CT study about femoral head osteonecrosis by Motomura et al. [22]. Another study by Ma et al. [38] showed that tissue ablation using high-intensity focused ultrasound ultimately led to osteonecrosis, which could be visualized by photopenic areas surrounded by an increased tracer region on planar scintigraphic images [20, 39]. The mechanism was based on a rapid temperature increase, which is comparable to cement hardening. Therefore, the results of the presented study add important information to the literature. All patients showed normal or high SPECT/CT tracer activity without osteonecrosis or a sclerotic rim, which would have been indicative of a necrotic-viable osseous junction.

In the knee, the value of SPECT has been appraised in a study by Hart et al. [40], who showed a significant correlation of SPECT findings with macroscopic cartilage configuration. The sensitivity rate of SPECT to exclude chondral degeneration was 97 %. Using SPECT/CTs, a cartilaginous lesion may be indirectly detected by an underlying osseous reaction. However, this may not be applied to the postoperative setting of the present study. Another study by Soininvaara et al. [41] showed that SPECT/CT is useful in the evaluation of bone remodeling after knee arthroplasty. They reported higher tracer uptake values at 12 months postoperatively with decreasing values between 12 and 24 months. The present study is consistent with this report, showing increased SPECT/CT activity in a lengthy period of time, although the general impression was a decrease in the mean uptake ratio with time. This increased activity can most likely be attributed to bone remodeling after surgical fracture treatment [38].

In the presented study, no postoperative infection or delayed wound healing was observed and all patients regained their pre-operative ability to work. The reasons for only three points at the Tegner and Lysholm score [24] and fair results at the Lysholm score [25] are multifactorial. The follow up period was not consistent in all cases, meaning some patients may have missed time to reach the pre-operative state. Furthermore, all but three patients scored good results at the Lysholm score. The mean patient age was rather advanced and two of the latter were the oldest patients, i.e. 85 years each, and severe medial gonarthrosis had already been present before receiving the surgical intervention presented herein. This is shown by the Tegner and Lysholm score, which did not change from the pre- to postoperative state. Indeed, the last patient, who did not score good results showed loss of reduction of the medial tibial plateau, which resulted in a knee arthroplasty. However, this patient suffered from a previously acquired ipsilateral hemineglect, which resulted in wheelchair ambulation and osteopenia, which may have prevented osseous consolidation. In retrospect, PMMA cement, which provides better stability at the initial postoperative phase, or a total knee arthroplasty may have been better alternatives than CaP to avoid secondary loss of correction. Taking all of the above into account, it may be believed that cement-augmented balloon tibioplasties seem to be associated with good clinical outcomes.

In a cadaveric study, Broome et al. [7] compared fracture reduction with an inflatable and a conventional metal tamp in fractures of six paired proximal tibias. They found the balloon to be superior in its minimally invasiveness and symmetric restoration of alignment of the joint surface. Its rounded appearance also mimics the lateral convexity of the tibia plateau very well. Another benefit over conventional tamps is the exact reduction because the balloon can be in- and deflated at a surgeon’s ease. This procedure proved to be safe without any perforation of the balloon or cement into the joint. Furthermore, they recommended a minimal distance of 3 mm from the balloon to the articular surface, but in our opinion, even five millimeters are sufficient enough to elevate the impacted fragment in order to avoid subchondral or chondral exothermic damage [10]. Evangelopoulos et al. [14] also reported good outcomes in seven atraumatic fractures of the tibial plateau in five osteoporotic patients. They highlighted the positive effect of minimal soft tissue damage and immediate full weight-bearing postoperatively by using pure PMMA. The fact that no periosteal stripping or fenestration was necessary may have led to fewer infections, less delayed wound healing, and pseudarthrosis. Another detailed clinical report about balloon tibioplasties by Pizanis et al. [12] described the successful application of this technique in five cases, where no postoperative complications were observed. We agree with their statement about the need for additional buttress plate or screw fixation in order to avoid sintering. Although fluoroscopy and arthroscopies are usually used to evaluate intraoperative reduction, CTs in the form of an O-arm can be added if necessary.

The limitations of this study include small sample size with consecutive inability to perform meaningful statistical analysis. Although MRI may directly visualize cartilaginous lesions, it is prone to metal-induced artifacts, hardly applicable in claustrophobic patients and not as sensitive in the identification of bony lesions, which is why one may prefer to use SPECT/CTs [42, 43]. However, in the future, MRI studies or SPECT/CT arthrography may be helpful in the direct assessment of cartilaginous vitality if metal artifacts can be minimized [44]. Furthermore, future studies may aim to compare viability of bone around PMMA and CaP bone cements in randomized clinical trials.

## Conclusions

This novel study about cement-augmented balloon tibioplasties showed that osseous vitality remains intact according to SPECT/CT analysis; irrespective of the type of cement. This procedure was safe and well-suited for lateral tibial plateau fractures in particular. Surgeons may take into account PMMA/HA bone cement for void filling in elderly patients with tibial plateau fractures without compromising bone viability.

## Abbreviations

(PMMA): Polymethyl methacrylate
(CaP): Calcium phosphate
(SPECT): Single photon emission computed tomography
(Tc): Technetium
(CT): Computed tomography
(MBq): Megabecquerel
(^99^mTc-DPD): ^99^mTc-3,3-diphosphono-1,2-propanodicarboxylic acid
(ROIs): Regions of interest
(HA): Hydroxyapatite
(SD): Standard deviation
(MRI): Magnetic resonance imaging
